# Supplementary material for: Consumer preference to utilise a mobile health app: A stated preference experiment
Source: PLoS One. 2020 Feb 21;15(2):e0229546. doi: 10.1371/journal.pone.0229546 (PMC7034842; doi:10.1371/journal.pone.0229546)
Supplement: S1 Appendix — (PDF) [file pone.0229546.s001.pdf]

APPENDIX 1. Orthogonal array- 9 dimensions with 4 levels

		Dimensions								
		**1**	**2**	**3**	**4**	**5**	**6**	**7**	**8**	**9**
**Choice sets**	**1)**	0	0	0	0	0	0	0	0	0
	**2)**	0	0	1	1	3	3	2	2	0
	**3)**	0	1	2	3	0	1	2	3	1
	**4)**	0	1	3	2	3	2	0	1	1
	**5)**	0	2	0	2	1	3	1	3	2
	**6)**	0	2	1	3	2	0	3	1	2
	**7)**	0	3	2	1	1	2	3	0	3
	**8)**	0	3	3	0	2	1	1	2	3
	**9)**	1	0	2	3	2	3	1	0	1
	**10)**	1	0	3	2	1	0	3	2	1
	**11)**	1	1	0	0	2	2	3	3	0
	**12)**	1	1	1	1	1	1	1	1	0
	**13)**	1	2	2	1	3	0	0	3	3
	**14)**	1	2	3	0	0	3	2	1	3
	**15)**	1	3	0	2	3	1	2	0	2
	**16)**	1	3	1	3	0	2	0	2	2
	**17)**	2	0	2	0	3	1	3	1	2
	**18)**	2	0	3	1	0	2	1	3	2
	**19)**	2	1	0	3	3	0	1	2	3
	**20)**	2	1	1	2	0	3	3	0	3
	**21)**	2	2	2	2	2	2	2	2	0
	**22)**	2	2	3	3	1	1	0	0	0
	**23)**	2	3	0	1	2	3	0	1	1
	**24)**	2	3	1	0	1	0	2	3	1
	**25)**	3	0	0	3	1	2	2	1	3
	**26)**	3	0	1	2	2	1	0	3	3
	**27)**	3	1	2	0	1	3	0	2	2
	**28)**	3	1	3	1	2	0	2	0	2
	**29)**	3	2	0	1	0	1	3	2	1
	**30)**	3	2	1	0	3	2	1	0	1
	**31)**	3	3	2	2	0	0	1	1	0
	**32)**	3	3	3	3	3	3	3	3	0

(Levels: 0=1st level, 1=2nd level, 2=3rd level, 3=4th level)
