# Supplementary material for: Consumer preference to utilise a mobile health app: A stated preference experiment
Source: PLoS One. 2020 Feb 21;15(2):e0229546. doi: 10.1371/journal.pone.0229546 (PMC7034842; doi:10.1371/journal.pone.0229546)
Supplement: S2 Appendix — (PDF) [file pone.0229546.s002.pdf]

APPENDIX 2. Page-by-page online survey

Welcome

We are inviting you to participate in a study exploring what factors might affect a person's willingness to download an app on a mobile phone. The app's main functionality is to collect personal information, that may help to ease the administrative burden often faced by medical centres.

The survey is going to ask you a series of hypothetical choice sets, each with slightly differing combinations of various factors. Each hypothetical choice set asks you to imagine that your local medical centre has recently expanded its range of health services and is looking for more ways to improve its services. After booking an appointment to see your doctor through their receptionist, you were asked by the receptionist to provide more information through an app downloadable on any smart phone.

This is student research that will count towards a Master's degree study, supervised by researchers based at Curtin University. If you would like to speak to someone about the study or this survey, please call Professor Suzanne Robinson on (08) 9266 4921. We hope that the project will allow us to add to the knowledge we have about consumers' preferences when utilising a mobile app for registration processes at medical facilities.

Your participation in this study is entirely voluntary. By completing the survey, you are voluntarily agreeing to participate. You can also choose to withdraw from the study at any time without any consequences. The survey software has been designed to protect your privacy and is secure. The survey is anonymous and confidential. No one will be able to identify you or your answers, and no one will know if you participated in the study or not. Your survey responses will be stored for the purposes of analysis, and combined with those from other participants and analysed as a group. Data from this research will be securely stored only on Curtin University's database and retained for 7 years.

Curtin University Human Research Ethics Committee (HREC) has approved this study (HREC approval number: HRE2018-0761). Should you wish to discuss the study with someone not directly involved, in particular, any matters concerning the conduct of the study or your rights as a participant, or you wish to make a confidential complaint, you may contact the Ethics Officer on (08) 9266 9223 or the Manager, Research Integrity on (08) 9266 7093 or email hrec@curtin.edu.au.

Disclosure

Your participation in this study is completely voluntary. You are not obliged to participate and may stop at any time. Your responses to this survey are strictly confidential and at no time will your answers be linked to your identity. This survey will take approximately 15-20 minutes to complete.

This survey has 4 sections

Section A contains background information about the questionnaire, a brief explanation of what to expect.

Section B contains eight different set choices. Your task is to understand a combination of nine factors from which you have to indicate your preferred choice for each set choice. There are no right or wrong answers.

Section C asks you some brief questions regarding your experience of the survey. This will help us improve similar questionnaires in future.

Section D contains questions about you, such as your education and medical history.

Are you willing to participate?

Select only one answer

- ○ Yes
- ○ No

Please select your gender:
Select only one answer

- o Male
- o Female
- o Other

Please select your age group:
Select only one answer

- o 18-24
- o 25-54
- o 55-64
- o 65+

Do you currently own a smartphone that you can use?
Select only one answer

- ○ Yes
- ○ No

Do you attend an appointment at a medical centre at least once a year?
Select only one answer

- ○ Yes
- ○ No

Section A: Background information for questionnaire (1/2)

You will soon be presented with a series of hypothetical choice sets, each with slightly differing combinations of various factors. Each hypothetical choice set asks you to imagine that your local medical centre has recently expanded its range of health services and is looking for more ways to improve its services. After booking an appointment to see your doctor through their receptionist, you were asked by the receptionist to provide more information through an app downloadable on any smart phone.

The information they are asking for, how the provided data can be used, and impact on registration with the app are all described in the following choice sets.

Your task will be to complete your choice on 8 different choice sets. These 8 choice sets have been selected randomly out from a bank of 32 different choice sets. Please read carefully through the following set options, and each set requires you to check ☒ your preferred answer to indicate whether you **would be willing**, or **would not be willing** to download the app and provide the information prior your appointment.

Please select only one answer per set question.

☐	I **would be willing** to download the app and provide the information before the appointment
☐	I **would not be willing** to download the app and provide the information before the appointment

Section A: Background information for questionnaire (2/2)

The main function of the app is to assist medical centres collect your information electronically prior to appointments. With your consent, registration staff at the medical centre will then be able to use the information you provided to pre-fill an electronic version of the documents required for appointment registration. This would replace the traditional paper-based registration process, potentially saving time for both you and the medical centre.

Having a range of allied health professionals at the medical centre, the same information you provide in the app may be used to pre-fill registration documentation for appointments with those other health professionals at your discretion. The app will be easily downloadable on to your smart phone via the app store free of charge.

Section B: Choice Sets

	Choice Set 1
**Registration Time**<br>Complete registration on the app will take approximately:	5 minutes
**Waiting Time**<br>It will reduce your waiting time for subsequent visits at the medical centre by approximately:	0 minutes
**Type of information**<br>It will be required of you to provide information on:	Your personal and family medical history and your medications
**Privacy**<br>Clinically relevant information that you provided on the app will be available to:	Your treating doctor attending to you
**Governance**<br>The storage and use of the information you provided will be guided with policies and regulations implemented by:	The medical centre
**Support**<br>Support staff will be available to help you register on the app through:	Face-to-face at the medical centre
**Convenience**<br>Upon complete registration on the app, it will give you the option to:	Book an appointment with your desired health professional
**Research**<br>The data you provided will be anonymized and may be used for research by:	Local universities
**Risk**<br>Upon complete registration, it **reduces** your risk of a medical error by:	0%

Select only one answer

☐	I **would be willing** to download the app and provide the information before the appointment
☐	I **would not be willing** to download the app and provide the information before the appointment

	Choice Set 2
**Registration Time**<br>Complete registration on the app will take approximately:	5 minutes
**Waiting Time**<br>It will reduce your waiting time for subsequent visits at the medical centre by approximately:	20 minutes
**Type of information**<br>It will be required of you to provide information on:	Your personal and family medical history, your medications, the number of tobacco products you smoke and the amount of alcohol you drink
**Privacy**<br>Clinically relevant information that you provided on the app will be available to:	Your treating doctor attending to you and other allied health professionals (ie. pharmacists, physiotherapists, clinicians and nurses)
**Governance**<br>The storage and use of the information you provided will be guided with policies and regulations implemented by:	The Australian Government
**Support**<br>Support staff will be available to help you register on the app through:	E-mail
**Convenience**<br>Upon complete registration on the app, it will give you the option to:	Does not offer any assistance with booking appointments
**Research**<br>The data you provided will be anonymized and may be used for research by:	Local universities
**Risk**<br>Upon complete registration, it **reduces** your risk of a medical error by:	10%

Select only one answer

☐	I **would be willing** to download the app and provide the information before the appointment
☐	I **would not be willing** to download the app and provide the information before the appointment

	Choice Set 3
**Registration Time**<br>Complete registration on the app will take approximately:	5 minutes
**Waiting Time**<br>It will reduce your waiting time for subsequent visits at the medical centre by approximately:	20 minutes
**Type of information**<br>It will be required of you to provide information on:	Your personal and family medical history, your medications, the number of tobacco products you smoke, the amount of alcohol you drink and the use of illicit drugs
**Privacy**<br>Clinically relevant information that you provided on the app will be available to:	Your treating doctor attending to you
**Governance**<br>The storage and use of the information you provided will be guided with policies and regulations implemented by:	A private consultancy firm
**Support**<br>Support staff will be available to help you register on the app through:	Over the phone
**Convenience**<br>Upon complete registration on the app, it will give you the option to:	Reschedule appointments in advance
**Research**<br>The data you provided will be anonymized and may be used for research by:	Private Pharmaceutical companies
**Risk**<br>Upon complete registration, it **reduces** your risk of a medical error by:	10%

Select only one answer

☐	I **would be willing** to download the app and provide the information before the appointment
☐	I **would not be willing** to download the app and provide the information before the appointment

	Choice Set 4
**Registration Time**<br>Complete registration on the app will take approximately:	10 minutes
**Waiting Time**<br>It will reduce your waiting time for subsequent visits at the medical centre by approximately:	10 minutes
**Type of information**<br>It will be required of you to provide information on:	Your personal and family medical history, your medications, the number of tobacco products you smoke and the amount of alcohol you drink
**Privacy**<br>Clinically relevant information that you provided on the app will be available to:	Your treating doctor attending to you, other doctors and allied health professionals (ie. pharmacists, physiotherapists, clinicians and nurses)
**Governance**<br>The storage and use of the information you provided will be guided with policies and regulations implemented by:	None
**Support**<br>Support staff will be available to help you register on the app through:	Face-to-face at the medical centre
**Convenience**<br>Upon complete registration on the app, it will give you the option to:	Book an appointment with your desired health professional
**Research**<br>The data you provided will be anonymized and may be used for research by:	No one
**Risk**<br>Upon complete registration, it **reduces** your risk of a medical error by:	10%

Select only one answer

☐	I **would be willing** to download the app and provide the information before the appointment
☐	I **would not be willing** to download the app and provide the information before the appointment

	Choice Set 5
**Registration Time**<br>Complete registration on the app will take approximately:	10 minutes
**Waiting Time**<br>It will reduce your waiting time for subsequent visits at the medical centre by approximately:	10 minutes
**Type of information**<br>It will be required of you to provide information on:	Your personal and family medical history, your medications, the number of tobacco products you smoke, the amount of alcohol you drink and the use of illicit drugs
**Privacy**<br>Clinically relevant information that you provided on the app will be available to:	Your treating doctor attending to you
**Governance**<br>The storage and use of the information you provided will be guided with policies and regulations implemented by:	The medical centre
**Support**<br>Support staff will be available to help you register on the app through:	No support available
**Convenience**<br>Upon complete registration on the app, it will give you the option to:	Send notification reminders about your next appointment on your smart phone
**Research**<br>The data you provided will be anonymized and may be used for research by:	Government researchers
**Risk**<br>Upon complete registration, it **reduces** your risk of a medical error by:	10%

Select only one answer

☐	I **would be willing** to download the app and provide the information before the appointment
☐	I **would not be willing** to download the app and provide the information before the appointment

	Choice Set 6
**Registration Time**<br>Complete registration on the app will take approximately:	30 minutes
**Waiting Time**<br>It will reduce your waiting time for subsequent visits at the medical centre by approximately:	0 minutes
**Type of information**<br>It will be required of you to provide information on:	Your personal and family medical history and your medications
**Privacy**<br>Clinically relevant information that you provided on the app will be available to:	Your treating doctor attending to you, other doctors, allied health professionals, the Australian Government and insurance companies
**Governance**<br>The storage and use of the information you provided will be guided with policies and regulations implemented by:	The Australian Government
**Support**<br>Support staff will be available to help you register on the app through:	E-mail
**Convenience**<br>Upon complete registration on the app, it will give you the option to:	Send notification reminders about your next appointment on your smart phone
**Research**<br>The data you provided will be anonymized and may be used for research by:	Government researchers
**Risk**<br>Upon complete registration, it **reduces** your risk of a medical error by:	10%

Select only one answer

☐	I **would be willing** to download the app and provide the information before the appointment
☐	I **would not be willing** to download the app and provide the information before the appointment

	Choice Set 7
**Registration Time**<br>Complete registration on the app will take approximately:	30 minutes
**Waiting Time**<br>It will reduce your waiting time for subsequent visits at the medical centre by approximately:	0 minutes
**Type of information**<br>It will be required of you to provide information on:	Your personal and family medical history, your medications and the number of tobacco products you smoke
**Privacy**<br>Clinically relevant information that you provided on the app will be available to:	Your treating doctor attending to you, other doctors, allied health professionals and the Australian Government
**Governance**<br>The storage and use of the information you provided will be guided with policies and regulations implemented by:	A private consultancy firm
**Support**<br>Support staff will be available to help you register on the app through:	Over the phone
**Convenience**<br>Upon complete registration on the app, it will give you the option to:	Book an appointment with your desired health professional
**Research**<br>The data you provided will be anonymized and may be used for research by:	No one
**Risk**<br>Upon complete registration, it **reduces** your risk of a medical error by:	10%

Select only one answer

☐	I **would be willing** to download the app and provide the information before the appointment
☐	I **would not be willing** to download the app and provide the information before the appointment

	Choice Set 8
**Registration Time**<br>Complete registration on the app will take approximately:	5 minutes
**Waiting Time**<br>It will reduce your waiting time for subsequent visits at the medical centre by approximately:	0 minutes
**Type of information**<br>It will be required of you to provide information on:	Your personal and family medical history and your medications
**Privacy**<br>Clinically relevant information that you provided on the app will be available to:	Your treating doctor attending to you
**Governance**<br>The storage and use of the information you provided will be guided with policies and regulations implemented by:	The medical centre
**Support**<br>Support staff will be available to help you register on the app through:	Face-to-face at the medical centre
**Convenience**<br>Upon complete registration on the app, it will give you the option to:	Book an appointment with your desired health professional
**Research**<br>The data you provided will be anonymized and may be used for research by:	Local universities
**Risk**<br>Upon complete registration, it **reduces** your risk of a medical error by:	0%

Select only one answer

☐	I **would be willing** to download the app and provide the information before the appointment
☐	I **would not be willing** to download the app and provide the information before the appointment

Section C: Your experience of the survey

How difficult was it for you to select your choice for each given set choice?
Select only one answer

Very difficult	Difficult	Neither easy nor difficult	Easy	Very easy
○	○	○	○	○

If not, please clearly state any issue(s) in the box below, including how the survey could be improved on.

Please be as detailed as possible in the box below.

Section D: More information about you

Which one of the following two geographic regions do you consider yourself currently living in?

Select only one answer and enter in your postcode in the box provided

- Metropolitan
- Rural

Please enter in your postcode: [ ]

What is your main language?

Select only one answer

- English
- Other: [ ]

Do you identify as an Aboriginal or Torres Strait Islander?

Select only one answer

- Yes
- No

Please select is the highest level of education you have completed.

Select only one answer

- Bachelor Degree level and above
- Advanced Diploma and Diploma level
- Certificate level III or IV
- Year 12
- Year 11 or below
- Not sure or refused

Which of the following categories best describes your annual gross (before tax) household income:

Select only one answer

- Less than $24,000 AUD
- $24,001 to $50,000
- $50,001 to $84,000
- $84,001 to $129,000
- More than $129,000
- Not sure or refused

Do you currently have any long-term medical conditions?

Select only one answer

- ○ Yes
- ○ No

If so, please specify the number of chronic conditions?

Please enter whole numerical digit(s)

- ○ ☐

Select as many as apply to you

- Heart disease(s)
- Dementia / Alzheimer’s disease
- Asthma / Chronic Obstructive Pulmonary Disease (COPD)
- Diabetes
- Kidney disease(s)
- Bone disease(s)
- Blood disease(s)
- Other(s): please specify [Please fill in 'None' in the box if you do not have any medical conditions] ________

Thank You for your time and participation

That concludes the survey! 😊
