# Supplementary material for: Consumer preference to utilise a mobile health app: A stated preference experiment
Source: PLoS One. 2020 Feb 21;15(2):e0229546. doi: 10.1371/journal.pone.0229546 (PMC7034842; doi:10.1371/journal.pone.0229546)
Supplement: Attachment — Submitted filename: Rebuttal Letter PONE-D-19-23759.docx [file pone.0229546.s003.docx]

Dear Editorial Manager,

Thank you for your additional points for revision with regards to our manuscript PONE-D-19-23759. We have gone through each reference list and have reviewed and updated each reference so that it now conforms to the referencing style for PLOS ONE publications. We have made the necessary corrections and now re-submitting it to you.

Yours sincerely,

David Lim

11/10/19
